# Feasibility of Double-Deployment Small-Diameter Covered Metallic Stent for Malignant Distal Biliary Obstruction (with Video)

**DOI:** 10.3390/diagnostics14192233

**Published:** 2024-10-07

**Authors:** Ryota Nakano, Hideyuki Shiomi, Mamiko Okamoto, Yuta Kawase, Kohei Yoshihara, Ryota Yoshioka, Shoki Kawata, Yukihisa Yuri, Tomoyuki Takashima, Nobuhiro Aizawa, Naoto Ikeda, Takashi Nishimura, Shinya Fukunishi, Hirayuki Enomoto

**Affiliations:** Division of Hepatobiliary and Pancreatic Disease, Department of Gastroenterology, Hyogo Medical University, 1-1 Mukogawa-cho, Nishinomiya 663-8501, Hyogo, Japan; ri-nakano@hyo-med.ac.jp (R.N.); hyougo21m@gmail.com (M.O.); yt-kawase@hyo-med.ac.jp (Y.K.); ko-yoshihara@hyo-med.ac.jp (K.Y.); ri-yoshioka@hyo-med.ac.jp (R.Y.); sh-kawata@hyo-med.ac.jp (S.K.); yu-yukihisa@hyo-med.ac.jp (Y.Y.); tomo0204@hyo-med.ac.jp (T.T.); aizawa-n@hyo-med.ac.jp (N.A.); nikeneko@hyo-med.ac.jp (N.I.); tk-nishimura@hyo-med.ac.jp (T.N.); sh-fukunishi@hyo-med.ac.jp (S.F.); enomoto@hyo-med.ac.jp (H.E.)

**Keywords:** ERCP, metallic stent, CSEMS, malignant distal biliary obstruction, cholecystitis, pancreatitis

## Abstract

Background/Objectives: Covered self-expandable metallic stents (CSEMS) are commonly used to treat malignant distal biliary obstructions. A 10-mm CSEMS carries the risk of obstructing the pancreatic and cystic duct orifices by adhering to the bile duct; therefore, postoperative pancreatitis and cholecystitis are reported to occur at a certain frequency. We have adopted a new drainage technique for malignant distal biliary obstruction called ‘‘double-slim SEMS stenting” (DSS), where two small-diameter CSEMS are placed side-by-side. We aimed to compare the efficacy and safety of biliary drainage using DSS with those of conventional CSEMS. Methods: In total, 50 patients who underwent endoscopic biliary drainage for malignant distal biliary obstructions between April 2019 and March 2022 at Hyogo Medical University Hospital were enrolled. Patients were divided into DSS and Conventional groups, and the technical success rate, clinical success rate, adverse events, success rate for reintervention, recurrent biliary obstruction (RBO) rate, and time to RBO (TRBO) were evaluated. Results: There were no significant differences in patient characteristics between the DSS (n = 20) and Conventional groups (n = 30). The technical and clinical success rates were 100% in the DSS group. The incidence of adverse events was not significantly different between the two groups (DSS/Conventional: 10.0% [2/20]/20.0% [6/30]) (*p* = 0.34). No acute cholecystitis was observed in the DSS group. The incidence rates of RBO were 30% (6/20) and 43% (13/30) in the DSS and Conventional groups, respectively (*p* = 0.92). The median TRBO in the DSS group was 378 days, while the TRBO in the Conventional group was 195 days (*p* = 0.03), resulting in significantly longer TRBO in the DSS group. Conclusions: DSS emerges as a viable and safe approach for biliary drainage in malignant distal biliary obstruction, demonstrating a lower incidence of adverse events and longer TRBO compared to conventional CSEMS.

## 1. Introduction

Malignant distal biliary obstruction is caused by various malignant tumors such as pancreatic cancer, distal bile duct cancer, gallbladder cancer, and lymph node metastasis in other organs. Often, these conditions are at an advanced stage and require prompt multidisciplinary treatment, such as surgical intervention or chemotherapy. Additionally, malignant distal biliary obstruction frequently leads to severe complications such as obstructive jaundice and cholangitis, necessitating immediate drainage.

Endoscopic biliary drainage is widely recognized as an effective treatment for malignant distal biliary obstruction. Stents used for biliary drainage are commonly categorized as plastic or metal, and studies have reported that metal stents have longer stent patency [1,2,3,4]. Covered self-expandable metallic stents (CSEMSs) are commonly used for biliary drainage, have been reported to have excellent stent patency [5,6], and are used because of their ease of removal during reintervention. In endoscopic biliary drainage for malignant distal biliary obstruction, a large diameter (10 mm) fully covered self-expandable metallic stent (SEMS) is widely used. These stents relieve biliary obstruction with a wide stent diameter and have a longer patency period [1,4,7]. However, a 10-mm CSEMS carries the risk of obstructing the pancreatic and cystic duct orifices by adhering to the bile duct; therefore, postoperative pancreatitis and cholecystitis are reported to occur at a high frequency [4,8,9].

Delayed chemotherapy or surgical intervention for background cancer or non-treatability due to tumor progression can occur because of acute pancreatitis or cholecystitis that develops after endoscopic biliary drainage. Therefore, the development of a more effective endoscopic biliary drainage method with fewer complications is required for the drainage of malignant distal biliary obstruction.

Recently, a slim-diameter (6 mm) CSEMS was developed for the biliary tract. This has been reported to be useful for drainage in which multiple slim CSEMS are placed through a large-diameter channel of a fluoroscope for hilar malignant biliary obstructions [10,11]. However, the usefulness of a slim CSEMS for malignant distal biliary obstruction has not yet been reported.

**Figure 1:**
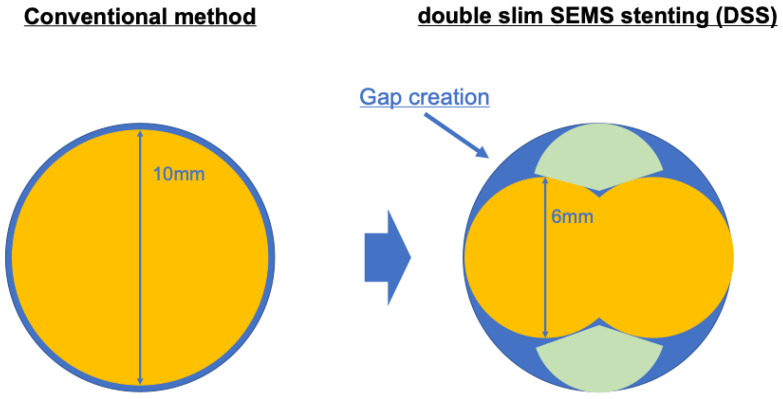
Schema diagram of DSS technique. DSS reduces the adhesion of stents to the bile duct by creating a gap between the two stents, thereby minimizing the risk of obstructing the pancreatic and gallbladder duct orifices.

We have developed a new drainage technique for malignant distal biliary obstruction called ‘‘double-slim SEMS stenting” (DSS), where two small-diameter CSEMS are placed side-by-side (Video S1). This technique reduces stent adhesion to the bile duct by creating a gap between the two stents, thereby minimizing the risk of obstructing the pancreatic and gallbladder duct orifices (Figure 1). In addition, because the stent diameter is equivalent to or greater than that of a conventional CSEMS, it is likely to achieve better patency and is considered an efficient and safe drainage method. Therefore, we aimed to compare the efficacy and safety of biliary drainage using DSS with those of conventional CSEMS.

## 2. Materials and Methods

### 2.1. Study Design

This study was a single-center retrospective cohort study. Between April 2019 and March 2022, patients with obstructive jaundice due to malignant distal biliary obstructions who met the following inclusion criteria were included. Malignant distal biliary obstruction was defined as biliary stenosis due to malignancy on the ampullary side, which is below the origin of the cystic duct of the extrahepatic bile duct, in reference to the classification of biliary tract cancers established by the Japanese Society of Hepato-Biliary-Pancreatic Surgery [12]. Inclusion criteria were (i) patients with malignant distal biliary obstruction for which endoscopic retrograde cholangiopancreatography (ERCP) was feasible; (ii) patients with a confirmed diagnosis of malignancy after pathological evaluation; (iii) patients with hepatic dysfunction, obstructive jaundice, or cholangitis associated with biliary insufficiency; and (iv) patients who are 20 years old or older at the time of consent. The following patients were excluded: Exclusion criteria were (i) patients with surgically altered gastrointestinal anatomy; (ii) patients who underwent biliary drainage with interventional endoscopic ultrasonography; (iii) patients whose biliary drainage with ERCP was hindered by tumor invasion into the duodenum,; (iv) patients whose endoscopic intervention was unfeasible owing to a decline in general health resulting from a primary ailment or severe serious complications in other organs; and (v) the patients who cannot provide consent to ERCP. Patients who met the inclusion criteria were divided into two groups: the DSS group, which underwent DSS, and the Conventional group, which underwent conventional SEMS placement. This study was conducted per the Ethical Guidelines for Medical and Health Research Involving Human Subjects and was approved by the ethics committees (no. 3431) of Hyogo Medical University (Hyogo, Japan). Consent for the acquisition of data was obtained on an opt-out basis. Relevant data were obtained from the patients’ medical records. All patients provided written informed consent prior to undergoing the endoscopic procedures.

### 2.2. Procedure

**Figure 2:**
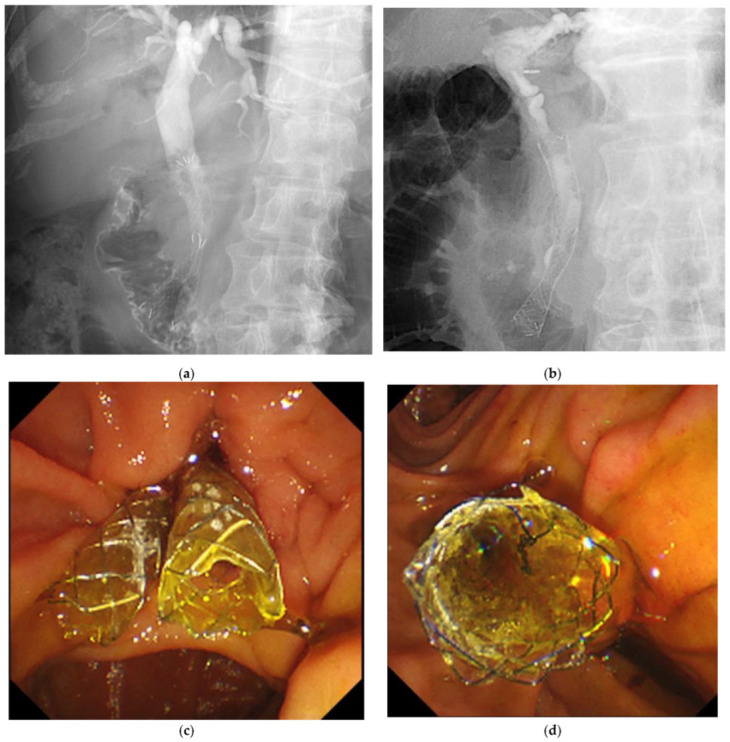
Procedure of deployment of DSS and Conventional groups. In the DSS group, two slim-diameter CSEMS with a diameter of 6 mm were positioned over the distal stenosis of the bile duct and released through the papilla (**a**,**b**). In the Conventional group, a single CSEMS with a wide diameter of 10 mm was positioned over the distal stenosis of the bile duct and released through the papilla (**c**,**d**).

All patients underwent endoscopic biliary drainage via ERCP and were admitted to the hospital on the day of the procedure. Antibiotics and appropriate fluid infusions were administered on the day of the procedure and the following day, respectively. In cases where endoscopic sphincterotomy (EST) had not been performed previously, EST was performed before CSEMS placement. All CSEMS were positioned over the distal bile duct stenosis and released through the papilla. In the DSS group, two slim-diameter CSEMS with a diameter of 6 mm (EGIS biliary stent, SB Kawasumi, Kanagawa, Japan) were deployed (Figure 2a,b), whereas in the Conventional group, a single CSEMS with a wider diameter of 10 mm (BONASTENT, Medico’s HIRATA, Osaka, Japan HIRZO STENTs, Zeon Medical INC., Tokyo, Japan; and Evolution Biliary Controlled-Release Stent, Cook Medical Japan G.K., Tokyo, Japan) was deployed (Figure 2c,d). The fluoroscope used for this procedure was a TJF-Q290V (Olympus, Tokyo, Japan). All cases were performed by a total of three endoscopists with at least 500 cases of ERCP experience as operators.

### 2.3. Procedure for DSS

**Figure 3:**
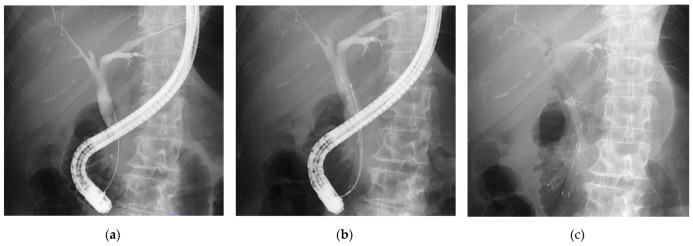
The detailed procedure of deployment of DSS. After bile duct cannulation, guidewires were sequentially inserted into the left and right intrahepatic bile ducts (**a**). Each guidewire was equipped with a 6-mm long and slim-diameter SEMS (**b**), and a CSEMS was inserted into one stent at a time using the side-by-side technique, with the CSEMS positioned over the distal stenosis of the bile duct and released through the papilla (**c**).

The DSS procedure was performed as follows: After bile duct cannulation with a wire-guided cannulation with contrast-assisted technique, each guidewire was inserted sequentially into the left and right intrahepatic bile ducts (Figure 3a). EST was performed on the ampulla of Vater. Each guidewire was equipped with a 6-mm slim-diameter SEMS, and a CSEMS was inserted into one stent at a time using the side-by-side technique (Figure 3b), with the CSEMS positioned over the distal stenosis of the bile duct and released through the papilla (Figure 3c).

### 2.4. Outcome Measurements

The study outcomes were technical success rate, clinical success rate, adverse events, success rate of reintervention, recurrent biliary obstruction (RBO) rate, and time to RBO (TRBO). Technical success was defined as the successful placement of a biliary stent at the intended bile duct site. Clinical success was defined as an improvement in the total bilirubin level by at least 50% or within the baseline range within 14 days of stenting. The severity of post-ERCP pancreatitis was classified according to the severity categories defined by Cotton et al. [13], while the severity of cholangitis was classified as mild, moderate, or severe based on the Tokyo Guidelines 2018 [14]. Successful reintervention was defined as a case in which the CSEMS was removed and replaced. TRBO was defined as the duration from the date of stenting to the date of RBO occurrence. RBO was characterized by stent occlusion or migration leading to hepatobiliary enzyme elevation or non-obstructive cholangitis necessitating reintervention. Overall survival was measured from the day of primary stent placement to the day of patient death.

### 2.5. Statistics Analysis

TRBO and overall survival were evaluated using the Kaplan–Meier method, and the DSS and Conventional groups were compared using the log-rank method. Censoring was used to account for patient death, surgery, and termination of follow-up before the occurrence of RBO during the evaluation. Reintervention for adverse events following stent placement, such as stent removal or additional treatment, was also included in censoring. The Mann–Whitney U test was used to compare continuous variables, and the χ2 test or Fisher’s exact test was used to compare categorical variables. Statistical analyses were performed using JMP Pro ^®^ version 17.2.0. Statistical significance was set at *p* < 0.05.

## 3. Results

### 3.1. Patient Characteristics

A total of 50 patients were enrolled, with 20 in the DSS group and 30 in the Conventional group. Table 1 shows the characteristics of the patients in each group. The median age was 72 years (range 58–89) in the DSS group and 71.5 years (range 49–94) in the Conventional group. The sex distribution was as follows: 9 males and 11 females in the DSS group and 19 males and 11 females in the Conventional group. In the DSS group, bile duct stricture occurred in 19 cases of pancreatic cancer and 1 case of cholangiocarcinoma. In contrast, in the Conventional group, it occurred in 27 cases of pancreatic cancer, 2 cases of cholangiocarcinoma, and 1 case of lymph node metastasis from gastric cancer. There were no significant differences between the two groups in the presence of tumor invasion of the choledochal duct (*p* = 0.52), dilation of the main pancreatic duct (*p* = 0.20), or tumor invasion of the duodenum (*p* = 0.23). There were also no significant differences between the two groups in terms of prior biliary drainage (*p* = 0.32) or frequency of cholangitis (*p* = 0.09) before ERCP.

### 3.2. Procedure Details

Table 2 shows a comparison of the procedure details between the DSS and Conventional groups. The technical success rate was 100% in the DSS (16/16) and Conventional (30/30) groups, with no difference between the two groups. The DSS group exhibited a sufficiently high treatment success rate.

There was no significant difference between the two groups in the method of bile duct cannulation or the prevalence of EST. The time of stents placement was 35.4 min in the DSS group and 34.2 min in the Conventional group (*p* = 0.61), which was almost equivalent.

### 3.3. Clinical Outcome

The clinical success rate was 100% in the DSS (20/20) and Conventional (30/30) groups, with all patients achieving successful stenting and improvement in jaundice. No significant differences were observed between the two groups (Table 3).

Adverse events were observed in 10.0% (2/20) of patients in the DSS group, including one case of pancreatitis and one case of liver abscess. In the Conventional group, 20.0% (6/30) of the patients had pancreatitis (two cases), cholecystitis (three cases), and pancreatic leakage (one case). The incidence of adverse events was not significantly different between the two groups; however, there was a trend toward fewer adverse events in the DSS group (*p* = 0.34) (Table 3). In the DSS group, acute cholecystitis was not considered an adverse event. The success rate of reintervention was successful in all patients in the DSS group (100%, 6/6) and in 92.3% (12/13) of patients in the Conventional group. There was no significant difference between the two groups (*p* = 0.48), but reintervention was successful in all patients in the DSS group (Table 3).

### 3.4. TRBO

The incidence rates of RBO were 30% (6/20) and 43% (13/30) in the DSS and Conventional groups, respectively. The incidence of RBO tended to be lower in the DSS group; however, there was no significant difference between the two groups (*p* = 0.34) (Table 4).

**Figure 4:**
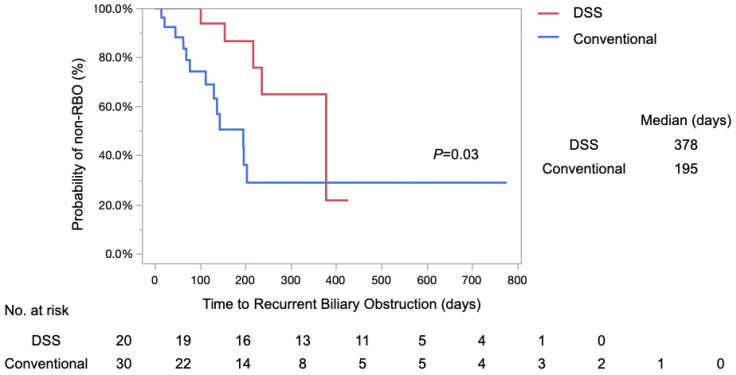
Kaplan–Meier analysis of the TRBO in the DSS and Conventional groups. The median TRBO in the DSS group was 378 days, while TRBO in the Conventional group was 195 days (*p* = 0.03, log-rank test). The rates of non-RBO at 3-, 6-, and 12 months post-stenting were found to be 100%, 86%, and 64% in the DSS group, and 74%, 57%, and 28% in the Conventional group, respectively.

A log-rank test using the Kaplan–Meier method revealed that the median TRBO in the DSS group was 378 days, whereas TRBO in the Conventional group was 195 days (*p* = 0.03), resulting in significantly longer TRBO in the DSS group (Figure 4).

The rates of non-RBO at 3, 6, and 12 months after stenting were 100%, 86%, and 64%, respectively, in the DSS group, and 74%, 50%, and 28%, respectively, in the Conventional group. The etiologies of RBO have been explored, revealing a range of underlying factors. In the DSS group, RBO caused by debris occlusion, stent migration to the distal side, and non-obstructive cholangitis accounted for two cases each, while one case of stent migration to the proximal side was noted. In contrast, the Conventional group had a higher incidence of stent occlusion caused by debris, with eight cases: two cases of stent migration to the distal side, one case of stent migration to the proximal side, and two cases of non-obstructive cholangitis (Table 4). None of the groups exhibited RBO resulting from tumor ingrowth.

### 3.5. Overall Survival

**Figure 5:**
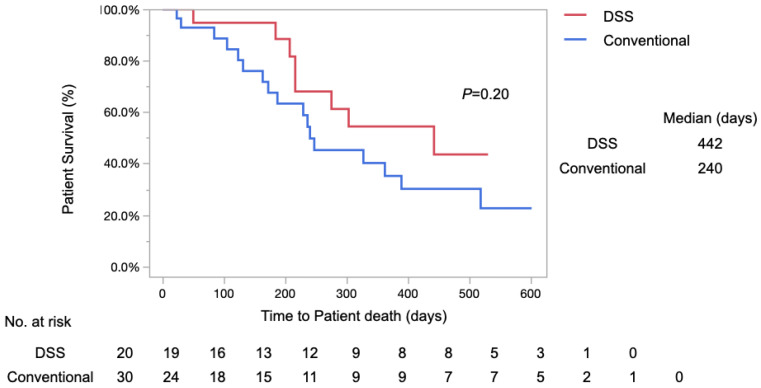
Kaplan–Meier analysis of the overall survival in the DSS and Conventional groups. The median survival was 442 days in the DSS group and was 240 days in the Conventional group (*p* = 0.20 log-rank test).

Overall survival was analyzed using the log-rank test and the Kaplan–Meier method (Figure 5). Median survival was 442 days in the DSS group and 240 days in the Conventional group. The 1-year survival rates were 54% and 35% in the DSS and Conventional groups, respectively. No significant difference in overall survival was observed between the two groups (*p* = 0.20).

## 4. Discussion

The DSS validated in this study is a new biliary drainage technique for obstructive jaundice due to malignant distal biliary obstruction, which allows long-term stent patency and safety with a lower adverse event rate. ERCP is the most commonly used drainage therapy for jaundice caused by malignant distal biliary obstructions. Several reports have demonstrated that metal biliary stents have longer patency than plastic biliary stents. Hence, metal stents are predominantly used in cases of unresectable malignant distal biliary obstruction that require long-term stenting [1,4,15]. The 6 mm diameter CSEMS used in this study is a newly developed biliary stent. In recent years, due to the development of devices, various attempts have been reported for the treatment of biliary and pancreatic malignant diseases, not only by endoscopic approaches, but also by surgical approaches [16].

In this study, the log-rank test using Kaplan–Meier curves for TRBO showed that the DSS group had significantly longer TRBO than the Conventional group (*p* = 0.03). Furthermore, the median TRBO was higher, at 378 days in the DSS group compared to 195 days in the Conventional group. These results suggest that DSS is a drainage method that can provide longer patency than conventional CSEMS. Given that previous reports of CSEMS have shown a TRBO of 154–285 days [4,17,18,19], the median TRBO of 378 days in the DSS group in this study was sufficiently favorable for stent patency compared to previous reports.

In this study, there was no significant difference in the time of stent placement between the DSS and Conventional groups (35.4 vs. 34.2 min; *p* = 0.61). DSS uses the side-by-side technique to insert two SEMS at one time, so the time required for stent insertion is comparable to that for conventional SEMS insertion. Since stent deployment takes less than 1 min per SEMS, the time of stent placement was not significantly different between the two groups.

In general, compared with uncovered SEMS, CSEMS have the advantages of no tumor ingrowth, easier stent removal and replacement in the event of stent occlusion, and longer stent patency duration [6]. However, the use of a CSEMS entails the risk of obstruction of the cholecystic and pancreatic ducts, and cholecystitis and pancreatitis have been reported to occur frequently following stenting [8,20,21,22,23]. Severe cases of cholecystitis and pancreatitis may necessitate prolonged fasting, infusion, and antibiotic therapies, which can force the patient to postpone treatment of the primary disease, raising concerns that optimal treatment may not be possible owing to tumor progression.

This study focused on the fact that, by deploying two slim-diameter (6 mm) CSEMS, the stent diameter is kept wider than that of a single CSEMS (10 mm), and the risk of obstruction of the cholecystic and pancreatic ducts is greatly reduced because of the creation of a gap between the two stents. In this study, the DSS group showed a lower, but not significant, incidence of total adverse events than the Conventional group (10.0% vs. 20.0%). Pancreatitis manifested in 5.0% (1/20) of cases in the DSS group and 6.6% (2/30) of cases in the Conventional group (*p* = 0.58), demonstrating similar occurrences in both cohorts. Conversely, cholecystitis was not observed in 0/16 patients in the DSS group, whereas it occurred in 10% (3/30) of patients in the Conventional group (*p* = 0.19). Post-ERCP pancreatitis is caused by impaired pancreatic juice outflow due to intraductal injection of contrast media or papillary edema caused by intubation stimulation, and pancreatic duct brush cytology and the pancreatic duct guidewire method have been reported as risks [24]. In this study, there were no significant differences between the two groups with respect to procedural details, such as cannulation method or EST. Pancreatitis noted in the DSS group was a case with naïve papilla and challenging biliary cannulation and was thought to be influenced by the cannulation technique, as these cases involved bile duct insertion using the pancreatic duct guidewire method. The two cases of pancreatitis that occurred in the Conventional group were both in the post-EST papilla and with plastic stent placement; however, they subsequently developed post-ERCP pancreatitis after CSEMS placement, suggesting that CSEMS placement was associated with the onset of pancreatitis.

The frequency of acute cholecystitis after CSEMS placement has been reported to be 3.5 to 6.6% [6,19]. Some reports indicate that CSEMS obstruction of the cholecystic duct is a risk factor for the development of cholecystitis [25,26,27]. Another reported risk factor includes tumor invasion into the cholecystic duct [28]; however, in this study, there was no difference in the number of patients with tumor invasion into the cholecystic duct between the two groups. Although the number of adverse events observed in this study was limited and requires confirmation through future extensive cohort studies, the findings suggest that DSS is a safer drainage technique than conventional CSEMS.

The cause of liver abscess in the DSS group was due to the migration of SEMS to the hilar side. The case showed an elevated inflammatory response without elevated biliary enzymes and was diagnosed as a liver abscess on computed tomography. In the DSS group, only one case of stent migration to the hilar side was observed, but there was no significant difference in the incidence of migration between the two groups. Further study with a larger number of patients is required to evaluate the incidence of migration.

Pancreatic cancer is the predominant cause of malignant distal biliary obstruction. In this study, the majority of participants had pancreatic cancer. While randomized controlled trials specifically targeting pancreatic cancer are limited, Kitano et al. highlighted that the mean duration of stent patency was 219 days when employing CSEMS compared to uncovered SEMS (UCSEMS) in cases of unresectable pancreatic cancer [19]. Recently, it has been reported that preoperative adjuvant chemotherapy for resectable pancreatic cancer significantly improves overall survival [29]. A similar pattern has been reported in another randomized controlled trial of resectable and borderline resectable pancreatic cancer [30]. These findings signify a growing population of patients undergoing preoperative adjuvant chemotherapy for pancreatic cancer. Consequently, establishing a safe and efficient biliary drainage approach for this treatment modality is challenging. While the available studies on biliary drainage specifically focused on patients undergoing preoperative adjuvant chemotherapy for pancreatic cancer are limited, most reports indicate that metal stents offer greater utility than plastic stents, exhibiting a lower incidence of biliary obstruction [2,31]. The DSS technique has exhibited prolonged stent patency, low rates of RBO, and a low incidence of adverse events. These findings strongly indicate the remarkable effectiveness of DSS as a biliary drainage method, ensuring enduring patency in cases of unresectable pancreatic cancer and serving as a safe and efficient drainage approach for resectable pancreatic cancer.

This study demonstrated the usefulness of a new drainage technique based on the unique and unprecedented idea of placing two 6-mm diameter CSEMS in the distal bile duct for malignant distal biliary obstruction. However, this study has limitations, including its single-center retrospective design and potential operator bias. However, there was no significant difference in the operator selection between the DSS and Conventional groups. Additionally, in terms of safety, which is a critical aspect of DSS, there have been relatively few comparative investigations of the occurrence of adverse events. Consequently, we did not detect any significant differences in the frequencies of adverse events. Despite these limitations, DSS was indicated to be a drainage method that reduced the incidence of cholecystitis and pancreatitis adverse events and significantly prolonged TRBO. Future prospective studies with larger patient cohorts are required to validate the usefulness of DSS.

## 5. Conclusions

Our study suggests that DSS is a useful and safe approach for biliary drainage of malignant distal biliary obstruction, with significantly longer TRBO and lower rates of adverse events than conventional CSEMS.

## Figures and Tables

**Table 1:** Clinical characteristics of patients enrolled in the DSS and Conventional groups.

	Total (n = 50)	DSS (n = 20)	Conventional (n = 30)	*p* Value
Age (years), median ±SD	72 ± 8.9	72 ± 7.4	71.5 ± 9.4	0.71
Gender (male/female)	28/22	9/11	19/11	0.20
BMI * (kg/m^2^), mean ± SD	20.2 ± 3.1	20.2 ± 3.5	19.9 ± 3.1	0.76
Cause of biliary obstruction				0.55
Pancreatic cancer	46	19	27	
Cholangiocarcinoma	3	1	2	
Lymph node metastasis	1	0	1	
Cystic duct invasion (yes/no)	8/42	4/16	4/26	0.52
MPD * dilation (yes/no)	35/15	12/8	23/7	0.20
Duodenum invasion (yes/no)	13/37	7/13	6/24	0.23
Length of stenosis (cm), mean ± SD	2.1 ± 0.7	1.9 ± 0.6	2.3 ± 0.7	0.15
Initial drainage (yes/no)	34/16	12/8	22/8	0.32
Prior Cholangitis (yes/no)	17/33	10/10	7/23	0.09
Total bilirubin (mg/dL), mean ± SD	4.0 ± 4.2	4.3 ± 3.3	3.7 ± 4.5	0.51
CRP (mg/dL), median ± SD	3.7 ± 4.0	4.4 ± 5.1	3.2 ± 3.5	0.31

Initial drainage: Patients who had undergone biliary drainage prior to SEMS deployments. Prior cholangitis: Patients who had concurrent cholangitis when the SEMS was deployed. * BMI, body mass index; MPD, main pancreatic duct.

**Table 2:** Details of the procedure in stent deployment in the DSS and Conventional groups.

	Total (n = 50)	DSS (n = 20)	Conventional (n = 30)	*p* Value
Technical success, (yes, %)	50 (100%)	20 (100%)	30 (100%)	1
Cannulation time, (min) mean ± SD	11.7 ± 9.9	10.5 ± 10.1	12.2 ± 9.9	0.60
Method of cannulation WGC ^†^ with contrast-assisted/pancreatic guidewire/precut	39/7/4	12/6/2	27/1/2	0.39
Addition of Endoscopic Sphincterotomy, (yes/no)	29/21	12/8	17/13	0.81
The time of stents placement, (min) mean ± SD	34.6 ± 15.3	35.4 ± 10.8	34.2 ± 17.7	0.61
Length of CSEMS *, (n) 6/7/8/9 cm	18/4/27/1	5/0/15/0	13/4/12/1	0.37

* CSEMS; covered self-expandable metallic stent. ^†^ WGC; wire-guided cannulation.

**Table 3:** Details of the clinical outcome in the DSS and Conventional groups.

	Total (n = 50)	DSS (n = 20)	Conventional (n = 30)	*p* Value
Clinical success, (yes, %)	50 (100%)	20 (100%)	30 (100%)	1
Adverse events, (n, %)	8 (17.3%)	2 (10.0%)	6 (20.0%)	0.34
Pancreatitis	3	1	2	
Cholecystitis	3	0	3	
Liver abscess	1	1	0	
Pancreatic leakage	1	0	1	
Success for re-intervention	18/19 (94.7%)	6/6 (100%)	12/13 (92.3%)	0.48

**Table 4:** Recurrent biliary obstruction in the DSS and Conventional groups.

	DSS (n = 20)	Conventional (n = 30)	*p* Value
**The incidence of RBO** * (n, %)	6 (30%)	13 (43%)	0.34
**non-RBO rate, 3/6/12 month** (%)	100%/86%/64%	74%/57%/28%	
**reasons for RBO, (n)**			
**occlusion**			
debris	2	8	0.14
food impaction	0	0	
overgrowth	0	0	
**migration**	3	3	0.59
**non-obstructive cholangitis**	2	2	0.67

* RBO; recurrent biliary obstruction.

## Data Availability

The datasets presented in this article are not readily available because the data are part of an ongoing study. Requests to access the datasets should be directed to hideshio0403@gmail.com.

## Supplementary Materials

The following supporting information can be downloaded at: https://zenodo.org/records/13619147, Video S1: The “double-slim SEMS stenting” (DSS) procedure.
